# A Multimedia E-Book—*A Story of Health*: Filling a Gap in Environmental Health Literacy for Health Professionals

**DOI:** 10.1289/EHP222

**Published:** 2016-08-01

**Authors:** Mark D. Miller, Maria Valenti, Ted Schettler, Brian Tencza

**Affiliations:** 1 Western States Pediatric Environmental Health Specialty Unit, University of California San Francisco, San Francisco, California, USA; 2 California Environmental Protection Agency, Oakland, California, USA; 3 Collaborative on Health and the Environment, Bolinas, California, USA; 4 Science and Environmental Health Network, Ames, Iowa, USA; 5 Environmental Medicine Branch, Division of Toxicology and Human Health Sciences, Agency for Toxic Substances and Disease Registry, Atlanta, Georgia, USA.

## Abstract

Narrative approaches and storytelling are emerging as powerful health promotion tools that can spark interest, increase understanding of determinants of health, and translate complex science. A Story of Health, a multimedia e-book with continuing education credits was designed to harness the power of storytelling to increase environmental health literacy. Health professionals are a key audience. They recognize that patients may be suffering from preventable illnesses of environmental origin but often feel ill-equipped to educate individuals and families about risks associated with common exposures. A Story of Health seeks to fill this gap and help readers develop the competencies they need in order to help patients make informed choices, reduce health risks, improve quality of life, and protect the environment. Americans rate nurses and medical doctors as having the highest honesty and ethical standards of all professions. These medical professionals can play a key role in changing patterns of patient behavior and influencing public policies. The e-book provides an easily accessible method of developing environmental health competency. The multimedia format with graphical interpretations allows for quick reviews of topics or for more in-depth analysis via links to additional resources. The CE evaluations have been overwhelmingly positive.

## Introduction

Narrative approaches and storytelling are emerging as powerful health promotion tools that can increase understanding of determinants of health and translate complex science (Dahlstrom 2014). Case-based learning has long been used in medical education. *A Story of Health* multimedia e-book with continuing education (CE) credits was designed to harness the power of storytelling to increase the environmental health literacy of health professionals, policy makers, and health advocates; encourage inclusion of anticipatory guidance in professional practice, and stimulate policy changes.

*A Story of Health* capitalizes on the narrative approach to teaching by using fictional stories to convey how multiple environmental factors interact with genetics to affect health across the life span. The first installment of the 150-page peer-reviewed e-book, which includes chapters on asthma (Brett’s story), developmental disabilities (Amelia’s story), and childhood leukemia (Stephen’s story), was released in 2015 and is available online without cost (http://wspehsu.ucsf.edu/for-clinical-professionals/training/a-story-of-health-a-multi-media-ebook/). Free CE credits are offered through the Centers for Disease Control and Prevention (CDC) and the Agency for Toxic Substances and Disease Registry (ATSDR). About two-thirds of the downloads are accompanied by CE registration suggesting that CE credits are an incentive for health professionals to read *A Story of Health*.

The stories explore influences of the natural, built, chemical, food, economic, and social environments on health across the life span—from conception to elder years. The individual stories reveal how these environments are further expressed through education, family structures, housing, nutrition, access to health care, social supports or stressors, and more. Collectively, these multi-level variables interact to create conditions conducive to health and wellness—or vulnerability and disease. Health promoting interventions from the individual level to the policy level are highlighted to encourage action.

## Improving Environmental Health Literacy through an Ecological Approach

*A Story of Health’*s ecological approach is central to the concept of environmental health literacy, a relatively new subdiscipline that “combines key principles and procedural elements from the fields of risk communication, health literacy, environmental health sciences (EHS), communications’ research and safety culture” (Finn and O’Fallon 2015). According to the Society for Public Health Education (SOPHE), the measure of environmental literacy is the capacity to understand how “people and societies relate to each other and to natural systems,” as well as the ability to “read, understand and act on information regarding the environment” (SOPHE 2015). Health literacy means the ability to “understand, evaluate, and act on oral, written, and visual health information in order to mitigate risk and live healthier lives” (SOPHE 2015). *A Story of Health* integrates these two concepts to help readers develop environmental health literacy. Finn and O’Fallon (2015) conclude that environmental health literacy can potentially lead to “greater understanding of specific risks, the reduction of exposures, and the improvement of health outcomes for individuals and communities.”

## Filling a Gap in Health Professionals’ Environmental Health Literacy

Health professionals are a key audience for *A Story of Health* because they are highly regarded. For example, Americans rate nurses and medical doctors as having the highest honesty and ethical standards of all professions (Riffkin 2014). They can play a key role in changing patterns of patient behavior as well as influencing public policies (Kreuter et al. 2000; Kovarik 2005). However, research shows that many health professionals feel ill-equipped to meet the needs of patients regarding environmental health anticipatory guidance or to inform public policy. In a 2011 review, Gehle et al. (2011) pointed out that environmental medicine is “largely omitted in the continuum of U.S. medical education,” which has been demonstrated in a variety of surveys of medical practitioners.

According to the American Academy of Pediatrics (AAP), “Parents of young children are intensely interested in the impact of the environment on their children’s health. They may look to their pediatrician for guidance about how to evaluate news reports about potential hazards in the air, water, and food” (AAP 2012). Pediatricians, however, report low self-efficacy in taking an environmental history and being able to follow-up on environmental concerns related to their patients’ health. In surveys conducted in New York, Wisconsin, Minnesota, and Michigan, > 1,000 pediatricians agreed that children are suffering preventable illnesses of environmental origin, but they feel ill-equipped to educate families about common exposures (Trasande et al. 2006a, 2006b, 2008). The authors concluded that “gaps persist in practitioner knowledge about environmental health nationwide and across disciplines,” and “significant demand exists for specialized centers of excellence that can evaluate environmental health concerns and for educational opportunities” (Trasande et al. 2006a, 2008, 2010). These conclusions were mirrored in a similar survey of 695 pediatricians, childcare specialists, and nurses conducted in northwest China, with respondents indicating they “have strong beliefs regarding the role of the environment in children’s health, and frequently identify affected children” (Trasande et al. 2014). However, “few are trained in environmental history taking or rate self-efficacy highly in managing common hazards” (Trasande et al. 2014).

A 2015 survey of more than 200 pediatric oncologists, fellows, and nurse practitioners also underscored the need for increased training about environmental health exposures related to cancer (Zachek et al. 2015). Although 88% of respondents reported receiving questions from families about environmental exposures and cancers, “a lack of comfort with these topics seems to have limited their discussions with families about the role of environmental exposures in childhood cancer” (Zachek et al. 2015). In addition, > 90% felt that more knowledge about associations between environmental exposures and childhood cancer would be helpful in addressing these issues with their patients (Zachek et al. 2015).

A recent national online survey of more than 2,500 fellows of the American Congress of Obstetricians and Gynecologists (ACOG) also revealed that routine guidance to patients on the health effects of environmental exposures was not a high priority (Stotland et al. 2014). Although more than three-quarters of the fellows agreed that they could reduce patient exposures to environmental health hazards by counseling patients, half reported that they rarely take an environmental health history, and < 20% reported routinely asking about common environmental exposures, including several known developmental toxicants with widespread exposures (Stotland et al. 2014).

Acquiring the knowledge and skills to counsel patients and families about the risks associated with exposure to environmental toxicants may be challenging for health care providers because of busy schedules, required continuing medical education in their specialties, and the relative scarcity of professional training about environmental health. *A Story of Health* provides an alternative method of developing environmental health competency for health care providers, as it can be easily accessed online and reviewed at the time and pace of one’s choosing. The multimedia format with graphical interpretations allows for quick reviews of topics, or more in-depth analysis via links and references to additional resources. Web-based medical education matches the efficacy of more traditional forms of delivery such as face-to-face conferences and lectures (Khatony et al. 2009; Maloney et al. 2011) without the time and financial costs associated with the latter. *A Story of Health* CE course compares very favorably to other environmental health courses offered by ATSDR/CDC, such as *Principles of Pediatric Environmental Health*, *Asbestos*, *and Polychlorinated Biphenyls* (*PCBs*) *Toxicity*. During a 1-year period, *A Story of Health* CE course received more than double the registrations of one of the most popular ATSDR/CDC courses.

## Topics and Themes

Although the fictional narratives in *A Story of Health* describe the lives of people with different diseases, several common themes resonate throughout the e-book:

Important environmental influences come from the natural, chemical, food, built, and social environments.
Although there are exceptions, most diseases, as well as good health, are the result of complex interactions between genes and multiple environmental influences.
Early-life experiences, particularly during critical windows of development, can have profound beneficial or detrimental lifelong effects, even into elder years.
Preventing disease and promoting health require actions and commitments from the individual, family, community, and society, as they are all interconnected.

*A Story of Health* team is currently developing the fourth story for the e-book on infertility and reproductive health—*Reiko and Toshio’s Story*—that echo these common themes.

## *A Story of Health*: Framework and Content Development

**Figure 1. A Story of Health Framework.**
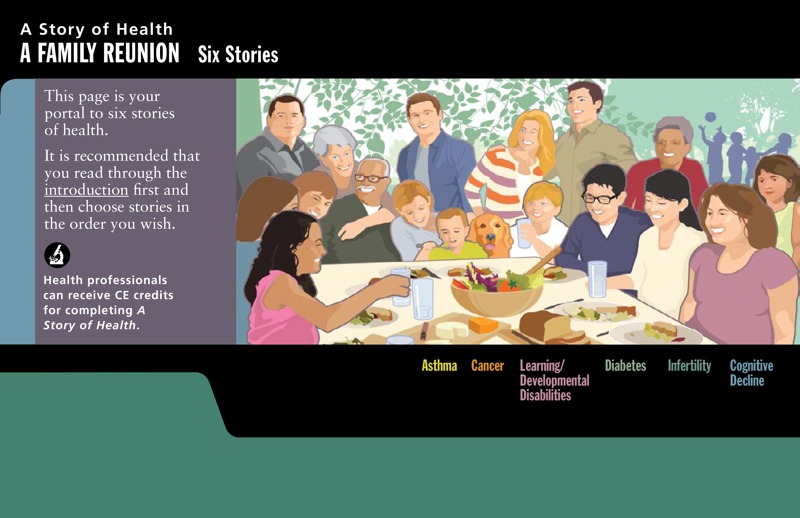
*A Story of Health* is designed to convey complex concepts about multi-level influences on health through a family reunion scenario. It sets the stage for stories to emerge about family members and friends who are experiencing a range of diseases and disorders. As the narratives unfold, the constellation of genetic and environmental circumstances that might contribute to disease are described based on current scientific understanding.

*A Story of Health* is designed to convey complex concepts about multi-level influences on health through a family reunion scenario (Figure 1). A nested ecological framework sets the stage for stories to emerge about family members who are experiencing a range of diseases and disorders. As the narratives unfold, the constellation of genetic and environmental circumstances that might contribute to disease are described based on current scientific understanding.

The fictional cases are communicated in text, illustrations, graphic images, videos, and links to additional resources and journal references. The stories include the following key concepts:

Early origins of childhood and adult disease
Epigenetics
Mechanisms of action
Allostatic load
Windows of susceptibility and opportunity
Effect modifiers
Environmental justice and health disparities

The stories weave in relevant information about disease trends and demographics. They also include potential interventions, policy recommendations, and helpful tools, such as environmental exposure checklists, for practical application in the real world.

The e-book draws content from the research of the top scientists in their fields and brings the collective expertise of the Pediatric Environmental Health Specialty Units network and the National Institute of Environmental Health Sciences Children’s Environmental Health Centers into the e-book in a variety of ways.

## Promotion

Promoting the availability of the e-book online via web sites, listservs, newsletters, YouTube presentations, and social media has been essential for reaching key audiences. *A Story of Health* was developed through a cooperative effort of the ATSDR, the Collaborative on Health and the Environment, the California EPA Office of Environmental Health Hazard Assessment, the Science and Environmental Health Network, and the Western States Pediatric Environmental Health Specialty Unit. Leveraging the resources and networks of all the partners has also been an important outreach strategy.

## Continuing Education Course Evaluations

Currently, more than 3,300 health professionals, including physicians, nurses, and health educators, have registered for the online course. Evaluations have been overwhelmingly affirming. In an analysis of responses from users in the second quarter of 2015, > 95% indicated that *A Story of Health* filled a gap in their skills or knowledge, and > 89% reported they plan to apply the new knowledge to develop strategies and interventions in their practices (Table 1).

**Table 1:** Continuing education feedback (April through June 2015) for three stories in *A Story of Health*: Brett’s story (asthma), Amelia’s story (development disabilities), and Stephen’s story (childhood leukemia).

Evaluation Feedback Questions	Strongly Agree + Agree = Total in %		
	Brett *N* = 225	Amelia *N* = 45	Stephen *N* = 34
1. The content and learning materials addressed a need or a gap in my knowledge or skills.	96	95	100
2. The difficulty level was appropriate.	97	98	97
3. The content expert(s) demonstrated expertise in the subject matter.	97	98	94
4. The delivery method used (e-learning etc.) was appropriate for the subject matter and helped me learn the content.	93	98	91
5. The instructional strategies (lecture, case scenarios, figures, tables, media, etc.) helped me learn the content.	95	98	94
6. Did you experience technical difficulties with this activity?	No 90	No 93	No 88
7. This activity effectively met my educational needs.	96	93	95
8. I will be able to apply the knowledge gained from this activity to increase or maintain my competence.	93	93	97
9. I will be able to apply the knowledge gained from this activity to my practice.	91	84	89
10. I will be able to apply the knowledge/skills gained from this activity to develop strategies/provide interventions.	89	91	89
11. I will be able to apply the knowledge gained from this activity to improve performance.	88	89	86
12. Do you anticipate barriers applying this knowledge?	No 93	No 93	No 91

## Next Steps

With additional funding, and with new CE evaluation tools being developed by the CDC, the authors hope to conduct follow-up surveys of those who have taken the CE course to further evaluate the impact of the e-book. Use the following links:

To download *A Story of Health*: http://wspehsu.ucsf.edu/for-clinical-professionals/training/a-story-of-health-a-multi-media-ebook/

To access CE registration: http://www.atsdr.cdc.gov/emes/health_professionals/index.html
